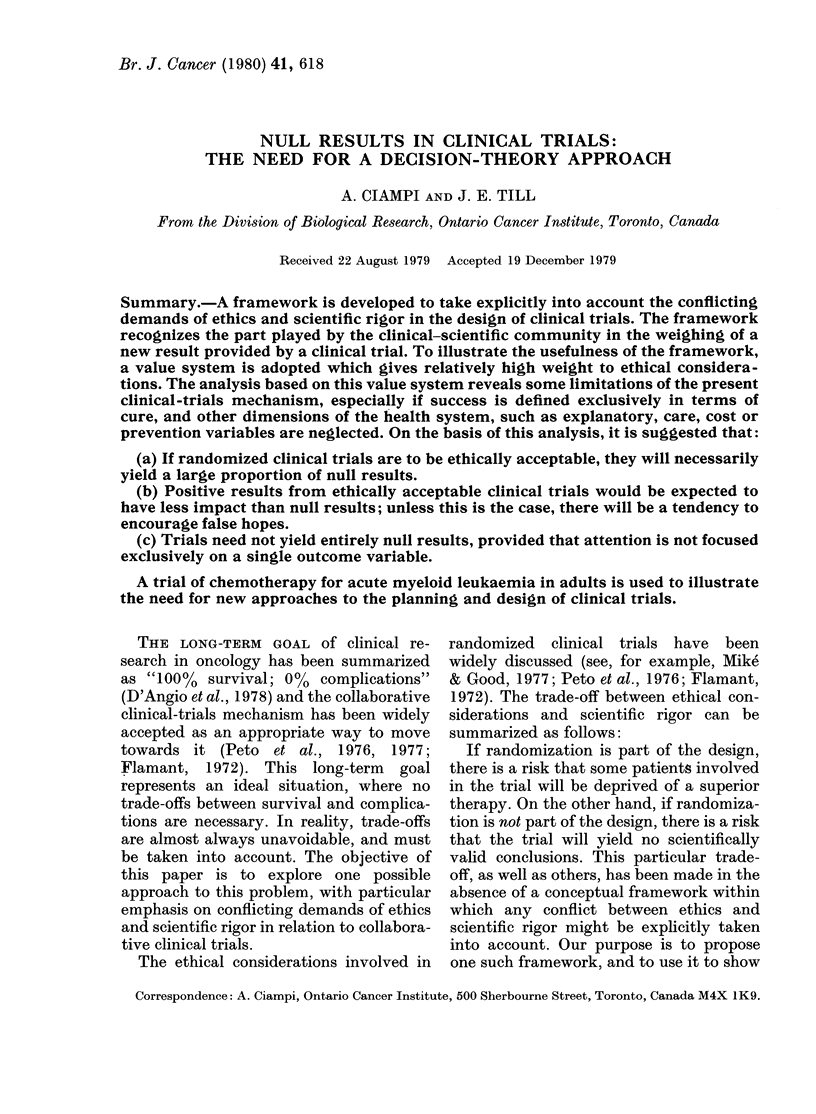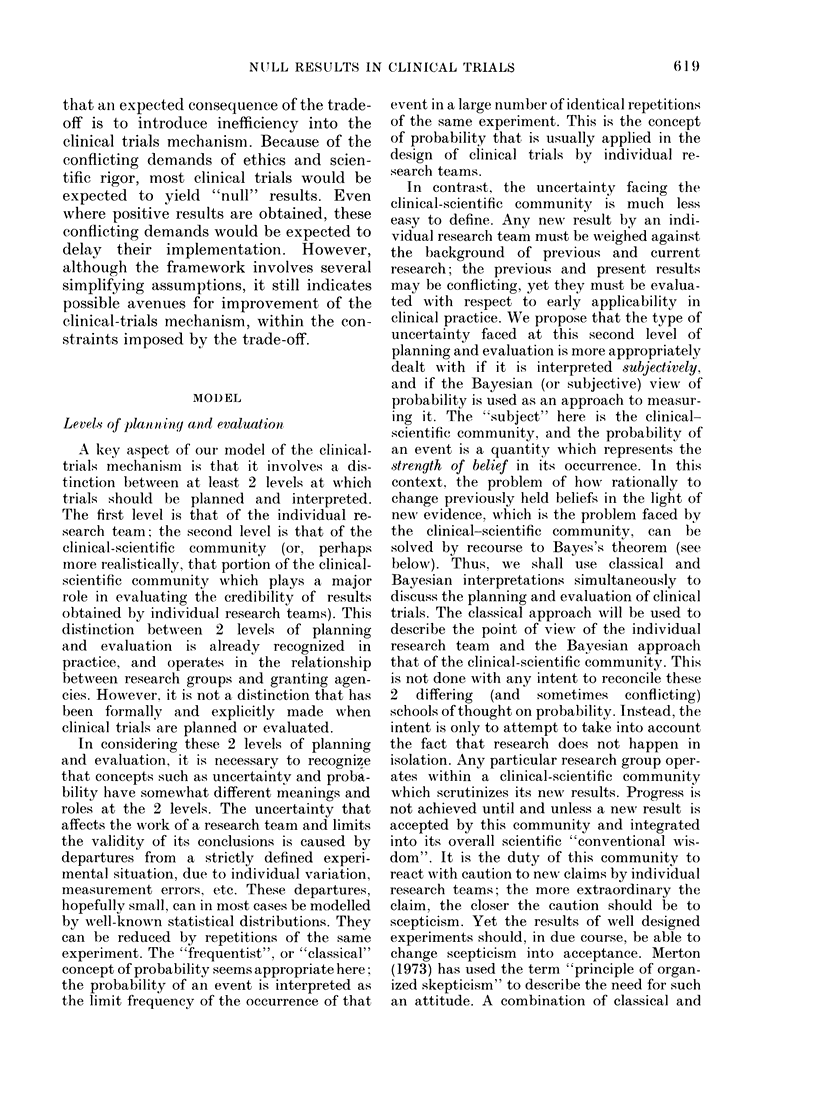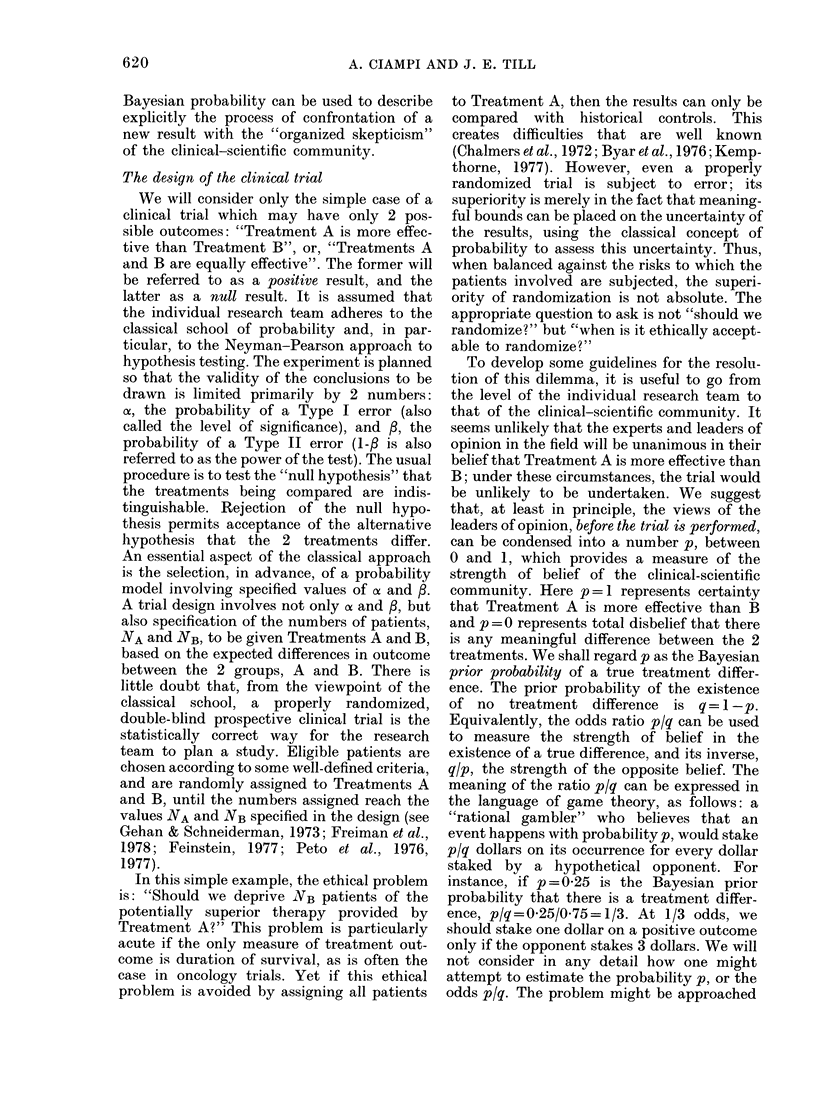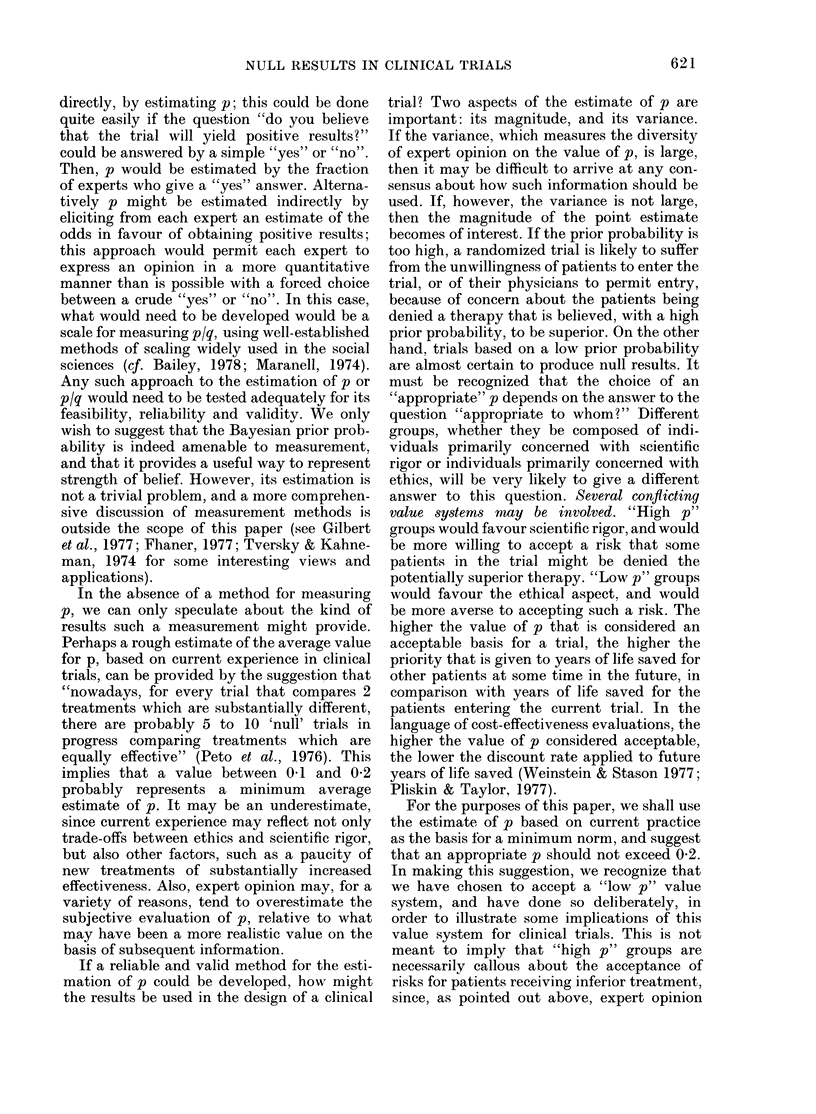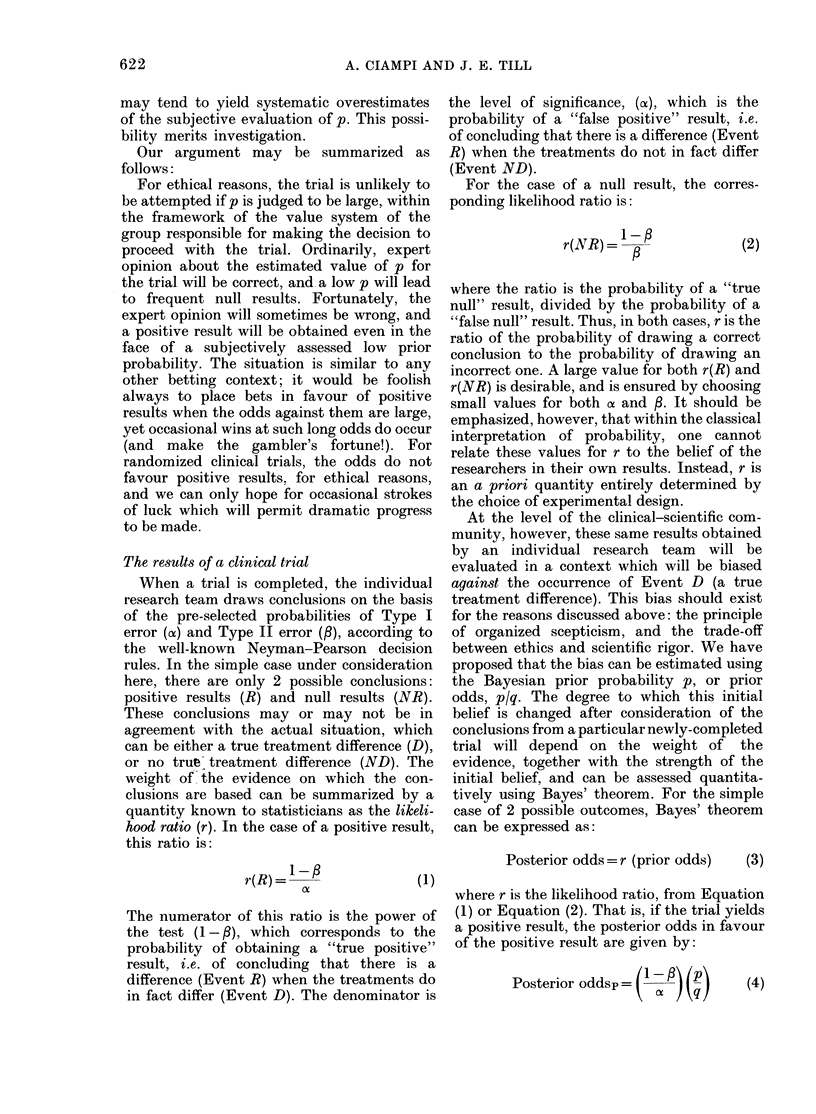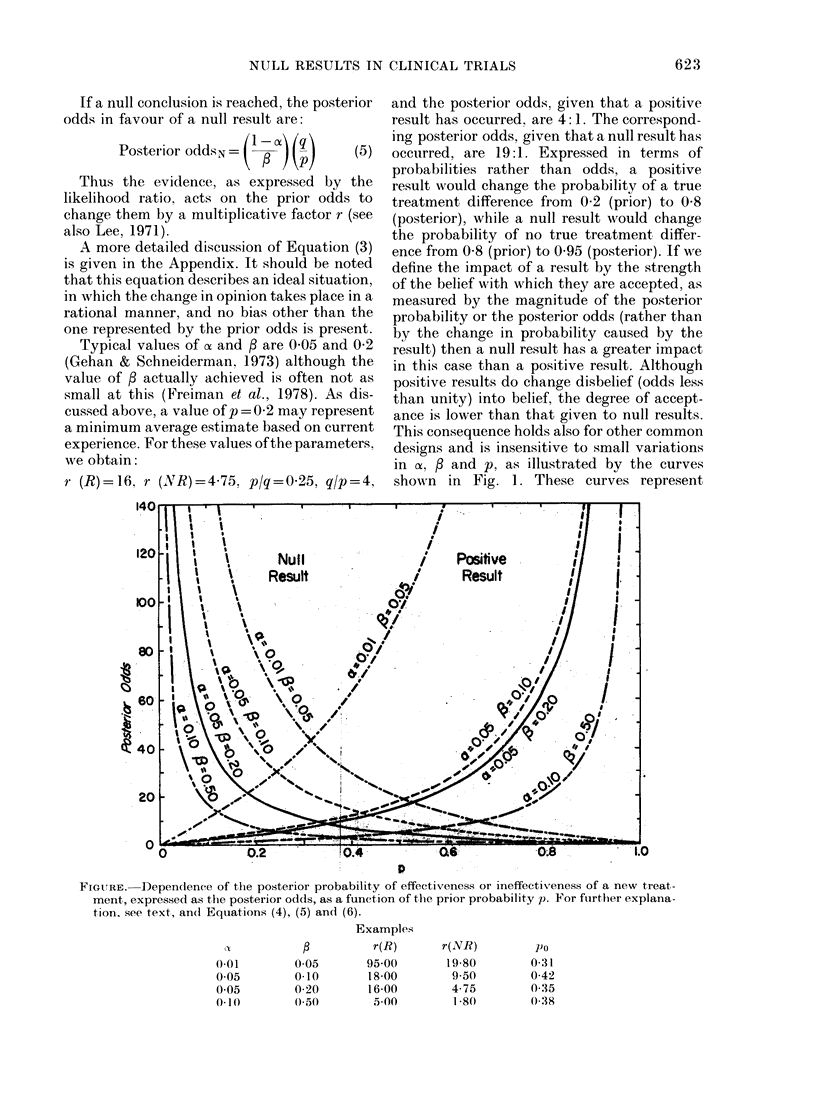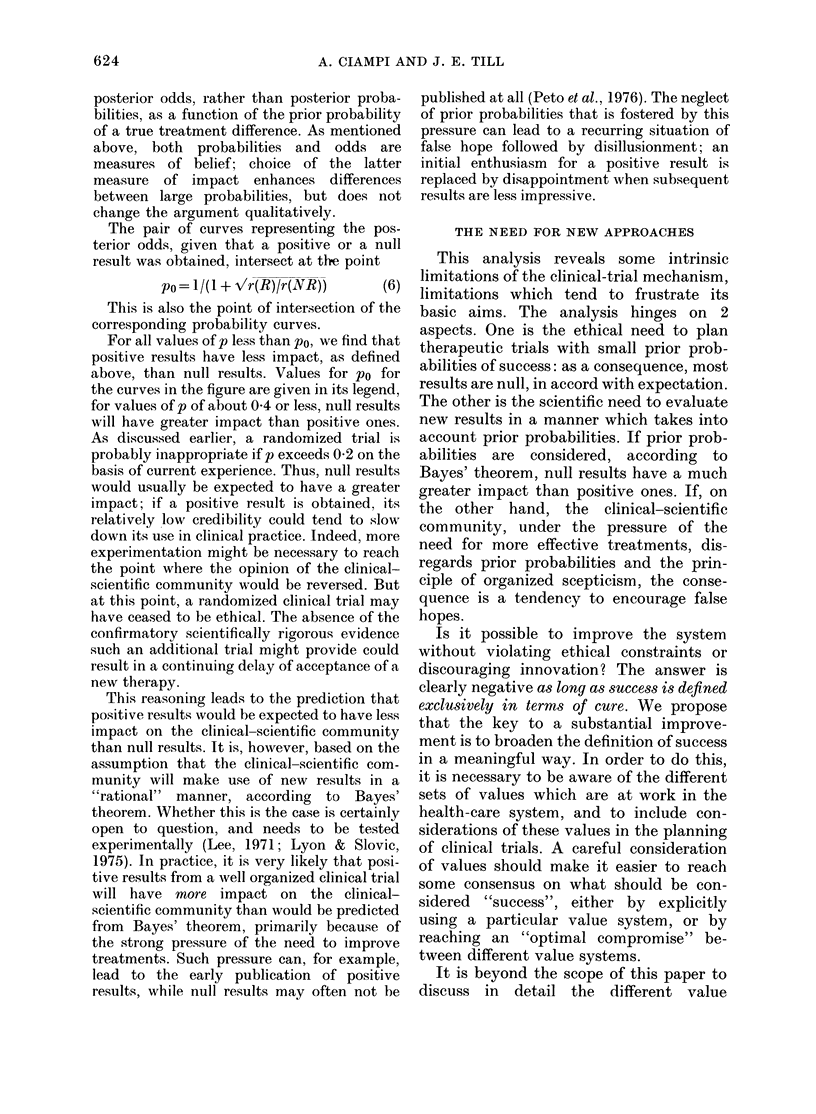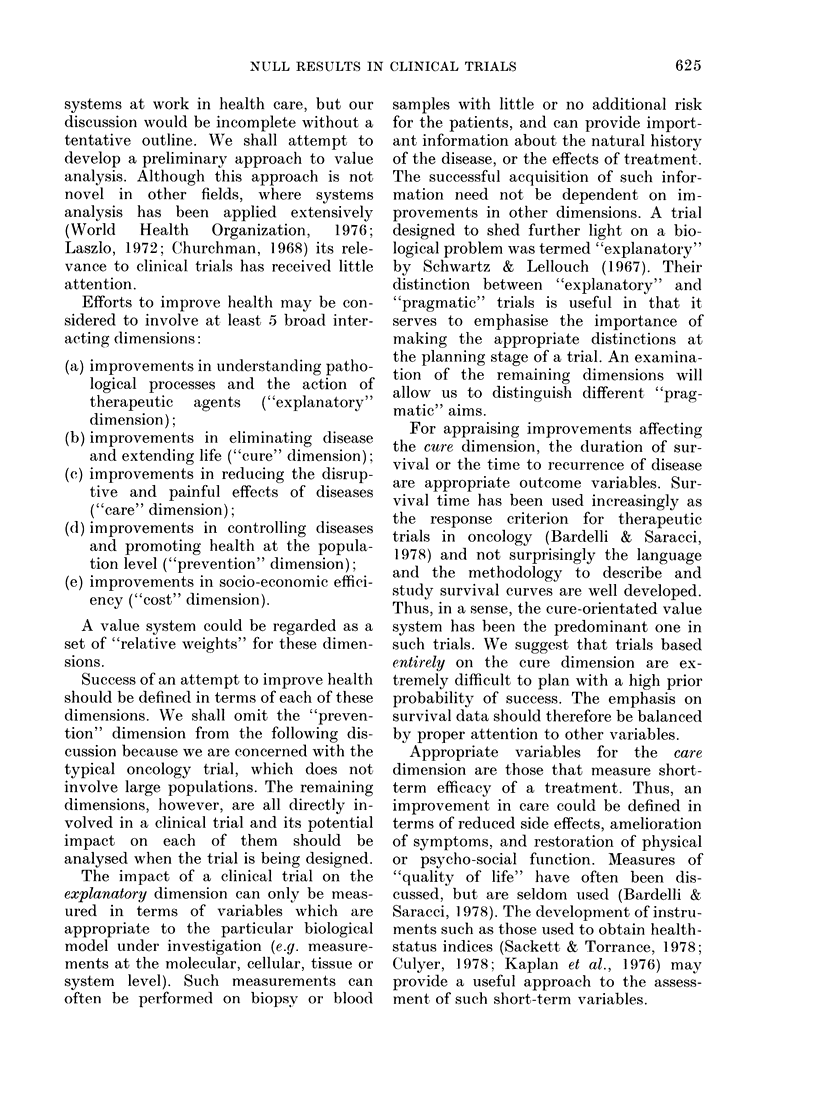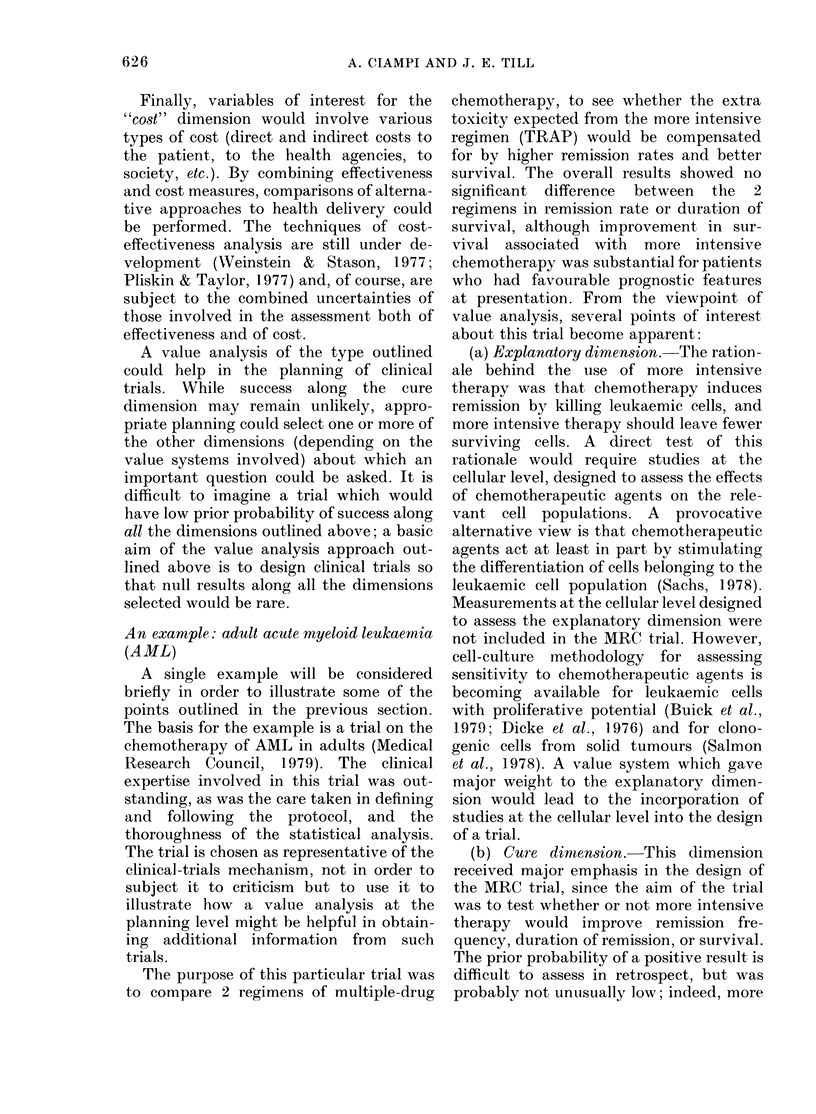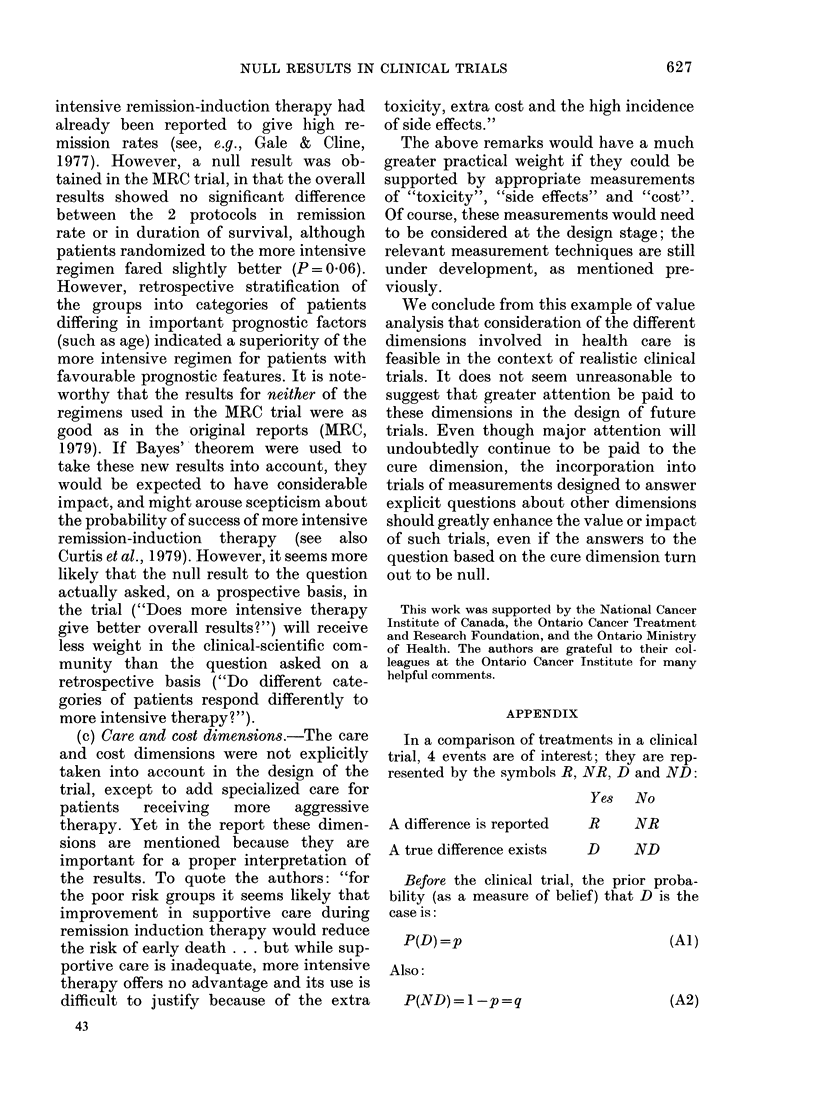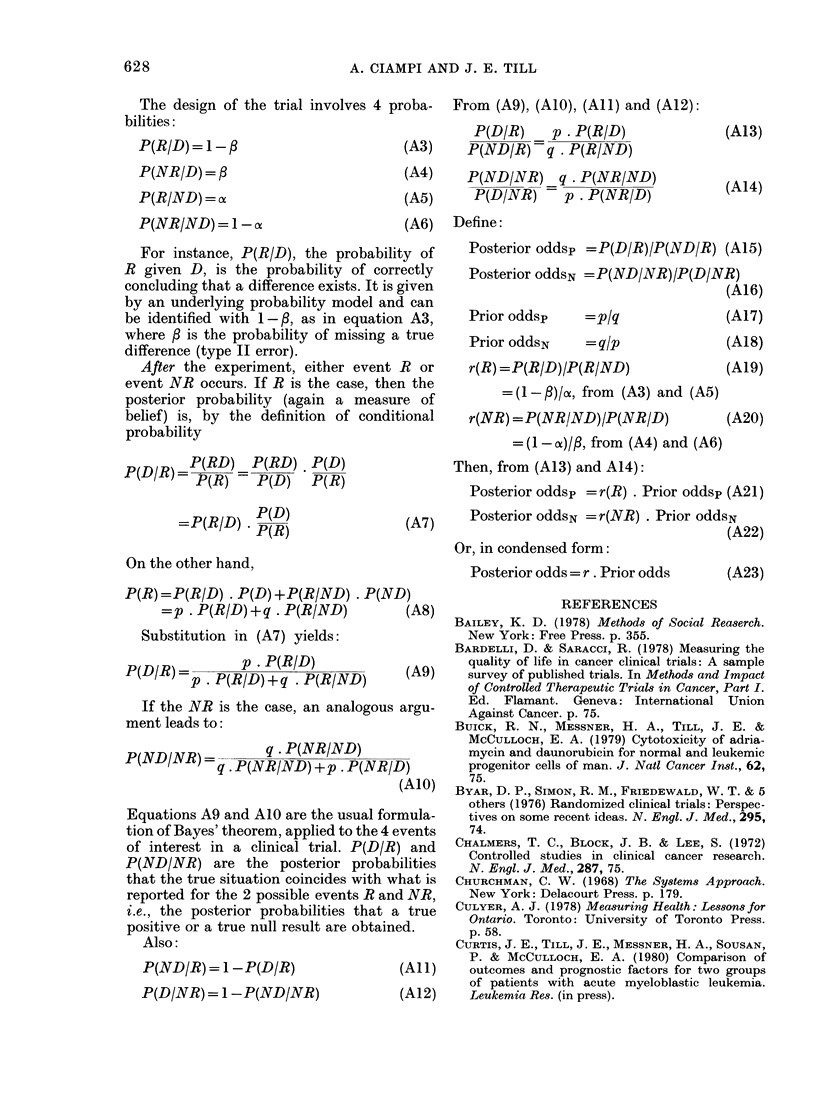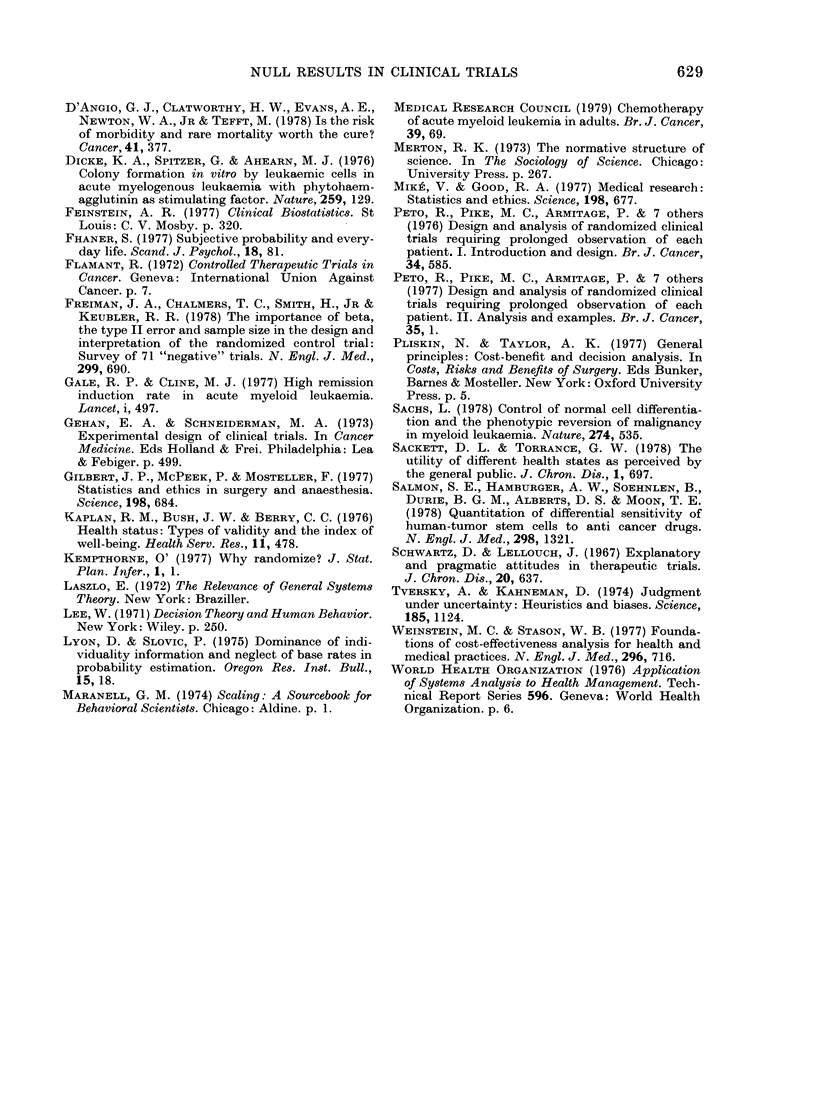# Null results in clinical trials: the need for a decision-theory approach.

**DOI:** 10.1038/bjc.1980.105

**Published:** 1980-04

**Authors:** A. Ciampi, J. E. Till

## Abstract

A framework is developed to take explicitly into account the conflicting demands of ethics and scientific rigor in the design of clinical trials. The framework recognizes the part played by the clinical-scientific community in the weighing of a new result provided by a clinical trial. To illustrate the usefulness of the framework, a value system is adopted which gives relatively high weight to ethical considerations. The analysis based on this value system reveals some limitations of the present clinical-trials mechanism, especially if success is defined exclusively in terms of cure, and other dimensions of the health system, such as explanatory, care, cost or prevention variables are neglected. On the basis of this analysis, it is suggested that: (a) If randomized clinical trials are to be ethically acceptable, they will necessarily yield a large proportion of null results. (b) Positive results from ethically acceptable clinical trials would be expected to have less impact than null results; unless this is the case, there will be a tendency to encourage false hopes. (c) Trials need not yield entirely null results, provided that attention is not focused exclusively on a single outcome variable. A trial of chemotherapy for acute myeloid leukaemia in adults is used to illustrate the need for new approaches to the planning and design of clinical trials.


*Br. J. Cancer* (1980) **41**, 618

### NULL RESULTS IN CLINICAL TRIALS: THE NEED FOR A DECISION-THEORY APPROACH

A. CIAMPI AND J. E. TILL

*From the Division of Biological Research, Ontario Cancer Institute, Toronto, Canada*

Received 22 August 1979 Accepted 19 December 1979

**Summary.—A framework is developed to take explicitly into account the conflicting demands of ethics and scientific rigor in the design of clinical trials. The framework recognizes the part played by the clinical–scientific community in the weighing of a new result provided by a clinical trial. To illustrate the usefulness of the framework, a value system is adopted which gives relatively high weight to ethical considerations. The analysis based on this value system reveals some limitations of the present clinical-trials mechanism, especially if success is defined exclusively in terms of cure, and other dimensions of the health system, such as explanatory, care, cost or prevention variables are neglected. On the basis of this analysis, it is suggested that:**

**(a) If randomized clinical trials are to be ethically acceptable, they will necessarily yield a large proportion of null results.**

**(b) Positive results from ethically acceptable clinical trials would be expected to have less impact than null results; unless this is the case, there will be a tendency to encourage false hopes.**

**(c) Trials need not yield entirely null results, provided that attention is not focused exclusively on a single outcome variable.**

**A trial of chemotherapy for acute myeloid leukaemia in adults is used to illustrate the need for new approaches to the planning and design of clinical trials.**

THE LONG-TERM GOAL of clinical research in oncology has been summarized as "100% survival; 0% complications" (D'Angio *et al.*, 1978) and the collaborative clinical-trials mechanism has been widely accepted as an appropriate way to move towards it (Peto *et al.*, 1976, 1977; Flamant, 1972). This long-term goal represents an ideal situation, where no trade-offs between survival and complications are necessary. In reality, trade-offs are almost always unavoidable, and must be taken into account. The objective of this paper is to explore one possible approach to this problem, with particular emphasis on conflicting demands of ethics and scientific rigor in relation to collaborative clinical trials.

The ethical considerations involved in randomized clinical trials have been widely discussed (see, for example, Miké & Good, 1977; Peto *et al.*, 1976; Flamant, 1972). The trade-off between ethical considerations and scientific rigor can be summarized as follows:

If randomization is part of the design, there is a risk that some patients involved in the trial will be deprived of a superior therapy. On the other hand, if randomization is *not* part of the design, there is a risk that the trial will yield no scientifically valid conclusions. This particular trade-off, as well as others, has been made in the absence of a conceptual framework within which any conflict between ethics and scientific rigor might be explicitly taken into account. Our purpose is to propose one such framework, and to use it to show

Correspondence: A. Ciampi, Ontario Cancer Institute, 500 Sherbourne Street, Toronto, Canada M4X 1K9.

that an expected consequence of the trade-off is to introduce inefficiency into the clinical trials mechanism. Because of the conflicting demands of ethics and scientific rigor, most clinical trials would be expected to yield "null" results. Even where positive results are obtained, these conflicting demands would be expected to delay their implementation. However, although the framework involves several simplifying assumptions, it still indicates possible avenues for improvement of the clinical-trials mechanism, within the constraints imposed by the trade-off.

### MODEL

### *Levels of planning and evaluation*

A key aspect of our model of the clinical-trials mechanism is that it involves a distinction between at least 2 levels at which trials should be planned and interpreted. The first level is that of the individual research team; the second level is that of the clinical-scientific community (or, perhaps more realistically, that portion of the clinical-scientific community which plays a major role in evaluating the credibility of results obtained by individual research teams). This distinction between 2 levels of planning and evaluation is already recognized in practice, and operates in the relationship between research groups and granting agencies. However, it is not a distinction that has been formally and explicitly made when clinical trials are planned or evaluated.

In considering these 2 levels of planning and evaluation, it is necessary to recognize that concepts such as uncertainty and probability have somewhat different meanings and roles at the 2 levels. The uncertainty that affects the work of a research team and limits the validity of its conclusions is caused by departures from a strictly defined experimental situation, due to individual variation, measurement errors, etc. These departures, hopefully small, can in most cases be modelled by well-known statistical distributions. They can be reduced by repetitions of the same experiment. The "frequentist", or "classical" concept of probability seems appropriate here; the probability of an event is interpreted as the limit frequency of the occurrence of that event in a large number of identical repetitions of the same experiment. This is the concept of probability that is usually applied in the design of clinical trials by individual research teams.

In contrast, the uncertainty facing the clinical-scientific community is much less easy to define. Any new result by an individual research team must be weighed against the background of previous and current research; the previous and present results may be conflicting, yet they must be evaluated with respect to early applicability in clinical practice. We propose that the type of uncertainty faced at this second level of planning and evaluation is more appropriately dealt with if it is interpreted *subjectively*, and if the Bayesian (or subjective) view of probability is used as an approach to measuring it. The "subject" here is the clinical–scientific community, and the probability of an event is a quantity which represents the *strength of belief* in its occurrence. In this context, the problem of how rationally to change previously held beliefs in the light of new evidence, which is the problem faced by the clinical–scientific community, can be solved by recourse to Bayes's theorem (see below). Thus, we shall use classical and Bayesian interpretations simultaneously to discuss the planning and evaluation of clinical trials. The classical approach will be used to describe the point of view of the individual research team and the Bayesian approach that of the clinical-scientific community. This is not done with any intent to reconcile these 2 differing (and sometimes conflicting) schools of thought on probability. Instead, the intent is only to attempt to take into account the fact that research does not happen in isolation. Any particular research group operates within a clinical-scientific community which scrutinizes its new results. Progress is not achieved until and unless a new result is accepted by this community and integrated into its overall scientific "conventional wisdom". It is the duty of this community to react with caution to new claims by individual research teams; the more extraordinary the claim, the closer the caution should be to scepticism. Yet the results of well designed experiments should, in due course, be able to change scepticism into acceptance. Merton (1973) has used the term "principle of organized skepticism" to describe the need for such an attitude. A combination of classical and

Bayesian probability can be used to describe explicitly the process of confrontation of a new result with the "organized skepticism" of the clinical–scientific community.

*The design of the clinical trial*

We will consider only the simple case of a clinical trial which may have only 2 possible outcomes: "Treatment A is more effective than Treatment B", or, "Treatments A and B are equally effective". The former will be referred to as a *positive* result, and the latter as a *null* result. It is assumed that the individual research team adheres to the classical school of probability and, in particular, to the Neyman–Pearson approach to hypothesis testing. The experiment is planned so that the validity of the conclusions to be drawn is limited primarily by 2 numbers: $\alpha$, the probability of a Type I error (also called the level of significance), and $\beta$, the probability of a Type II error ($1\text{-}\beta$ is also referred to as the power of the test). The usual procedure is to test the "null hypothesis" that the treatments being compared are indistinguishable. Rejection of the null hypothesis permits acceptance of the alternative hypothesis that the 2 treatments differ. An essential aspect of the classical approach is the selection, in advance, of a probability model involving specified values of $\alpha$ and $\beta$. A trial design involves not only $\alpha$ and $\beta$, but also specification of the numbers of patients, $N_A$ and $N_B$, to be given Treatments A and B, based on the expected differences in outcome between the 2 groups, A and B. There is little doubt that, from the viewpoint of the classical school, a properly randomized, double-blind prospective clinical trial is the statistically correct way for the research team to plan a study. Eligible patients are chosen according to some well-defined criteria, and are randomly assigned to Treatments A and B, until the numbers assigned reach the values $N_A$ and $N_B$ specified in the design (see Gehan & Schneiderman, 1973; Freiman *et al.*, 1978; Feinstein, 1977; Peto *et al.*, 1976, 1977).

In this simple example, the ethical problem is: "Should we deprive $N_B$ patients of the potentially superior therapy provided by Treatment A?" This problem is particularly acute if the only measure of treatment outcome is duration of survival, as is often the case in oncology trials. Yet if this ethical problem is avoided by assigning all patients to Treatment A, then the results can only be compared with historical controls. This creates difficulties that are well known (Chalmers *et al.*, 1972; Byar *et al.*, 1976; Kempthorne, 1977). However, even a properly randomized trial is subject to error; its superiority is merely in the fact that meaningful bounds can be placed on the uncertainty of the results, using the classical concept of probability to assess this uncertainty. Thus, when balanced against the risks to which the patients involved are subjected, the superiority of randomization is not absolute. The appropriate question to ask is not "should we randomize?" but "when is it ethically acceptable to randomize?"

To develop some guidelines for the resolution of this dilemma, it is useful to go from the level of the individual research team to that of the clinical–scientific community. It seems unlikely that the experts and leaders of opinion in the field will be unanimous in their belief that Treatment A is more effective than B; under these circumstances, the trial would be unlikely to be undertaken. We suggest that, at least in principle, the views of the leaders of opinion, *before the trial is performed*, can be condensed into a number $p$, between 0 and 1, which provides a measure of the strength of belief of the clinical-scientific community. Here $p=1$ represents certainty that Treatment A is more effective than B and $p=0$ represents total disbelief that there is any meaningful difference between the 2 treatments. We shall regard $p$ as the Bayesian *prior probability* of a true treatment difference. The prior probability of the existence of no treatment difference is $q=1-p$. Equivalently, the odds ratio $p/q$ can be used to measure the strength of belief in the existence of a true difference, and its inverse, $q/p$, the strength of the opposite belief. The meaning of the ratio $p/q$ can be expressed in the language of game theory, as follows: a "rational gambler" who believes that an event happens with probability $p$, would stake $p/q$ dollars on its occurrence for every dollar staked by a hypothetical opponent. For instance, if $p=0{\cdot}25$ is the Bayesian prior probability that there is a treatment difference, $p/q=0{\cdot}25/0{\cdot}75=1/3$. At 1/3 odds, we should stake one dollar on a positive outcome only if the opponent stakes 3 dollars. We will not consider in any detail how one might attempt to estimate the probability $p$, or the odds $p/q$. The problem might be approached directly, by estimating $p$; this could be done quite easily if the question "do you believe that the trial will yield positive results?" could be answered by a simple "yes" or "no". Then, $p$ would be estimated by the fraction of experts who give a "yes" answer. Alternatively $p$ might be estimated indirectly by eliciting from each expert an estimate of the odds in favour of obtaining positive results; this approach would permit each expert to express an opinion in a more quantitative manner than is possible with a forced choice between a crude "yes" or "no". In this case, what would need to be developed would be a scale for measuring $p/q$, using well-established methods of scaling widely used in the social sciences (*cf.* Bailey, 1978; Maranell, 1974). Any such approach to the estimation of $p$ or $p/q$ would need to be tested adequately for its feasibility, reliability and validity. We only wish to suggest that the Bayesian prior probability is indeed amenable to measurement, and that it provides a useful way to represent strength of belief. However, its estimation is not a trivial problem, and a more comprehensive discussion of measurement methods is outside the scope of this paper (see Gilbert *et al.*, 1977; Fhaner, 1977; Tversky & Kahneman, 1974 for some interesting views and applications).

In the absence of a method for measuring $p$, we can only speculate about the kind of results such a measurement might provide. Perhaps a rough estimate of the average value for p, based on current experience in clinical trials, can be provided by the suggestion that "nowadays, for every trial that compares 2 treatments which are substantially different, there are probably 5 to 10 'null' trials in progress comparing treatments which are equally effective" (Peto *et al.*, 1976). This implies that a value between 0·1 and 0·2 probably represents a minimum average estimate of $p$. It may be an underestimate, since current experience may reflect not only trade-offs between ethics and scientific rigor, but also other factors, such as a paucity of new treatments of substantially increased effectiveness. Also, expert opinion may, for a variety of reasons, tend to overestimate the subjective evaluation of $p$, relative to what may have been a more realistic value on the basis of subsequent information.

If a reliable and valid method for the estimation of $p$ could be developed, how might the results be used in the design of a clinical trial? Two aspects of the estimate of $p$ are important: its magnitude, and its variance. If the variance, which measures the diversity of expert opinion on the value of $p$, is large, then it may be difficult to arrive at any consensus about how such information should be used. If, however, the variance is not large, then the magnitude of the point estimate becomes of interest. If the prior probability is too high, a randomized trial is likely to suffer from the unwillingness of patients to enter the trial, or of their physicians to permit entry, because of concern about the patients being denied a therapy that is believed, with a high prior probability, to be superior. On the other hand, trials based on a low prior probability are almost certain to produce null results. It must be recognized that the choice of an "appropriate" $p$ depends on the answer to the question "appropriate to whom?" Different groups, whether they be composed of individuals primarily concerned with scientific rigor or individuals primarily concerned with ethics, will be very likely to give a different answer to this question. *Several conflicting value systems may be involved.* "High $p$" groups would favour scientific rigor, and would be more willing to accept a risk that some patients in the trial might be denied the potentially superior therapy. "Low $p$" groups would favour the ethical aspect, and would be more averse to accepting such a risk. The higher the value of $p$ that is considered an acceptable basis for a trial, the higher the priority that is given to years of life saved for other patients at some time in the future, in comparison with years of life saved for the patients entering the current trial. In the language of cost-effectiveness evaluations, the higher the value of $p$ considered acceptable, the lower the discount rate applied to future years of life saved (Weinstein & Stason 1977; Pliskin & Taylor, 1977).

For the purposes of this paper, we shall use the estimate of $p$ based on current practice as the basis for a minimum norm, and suggest that an appropriate $p$ should not exceed 0·2. In making this suggestion, we recognize that we have chosen to accept a "low $p$" value system, and have done so deliberately, in order to illustrate some implications of this value system for clinical trials. This is not meant to imply that "high $p$" groups are necessarily callous about the acceptance of risks for patients receiving inferior treatment, since, as pointed out above, expert opinion may tend to yield systematic overestimates of the subjective evaluation of $p$. This possibility merits investigation.

Our argument may be summarized as follows:

For ethical reasons, the trial is unlikely to be attempted if $p$ is judged to be large, within the framework of the value system of the group responsible for making the decision to proceed with the trial. Ordinarily, expert opinion about the estimated value of $p$ for the trial will be correct, and a low $p$ will lead to frequent null results. Fortunately, the expert opinion will sometimes be wrong, and a positive result will be obtained even in the face of a subjectively assessed low prior probability. The situation is similar to any other betting context; it would be foolish always to place bets in favour of positive results when the odds against them are large, yet occasional wins at such long odds do occur (and make the gambler's fortune!). For randomized clinical trials, the odds do not favour positive results, for ethical reasons, and we can only hope for occasional strokes of luck which will permit dramatic progress to be made.

*The results of a clinical trial*

When a trial is completed, the individual research team draws conclusions on the basis of the pre-selected probabilities of Type I error ($\alpha$) and Type II error ($\beta$), according to the well-known Neyman–Pearson decision rules. In the simple case under consideration here, there are only 2 possible conclusions: positive results ($R$) and null results ($NR$). These conclusions may or may not be in agreement with the actual situation, which can be either a true treatment difference ($D$), or no true treatment difference ($ND$). The weight of the evidence on which the conclusions are based can be summarized by a quantity known to statisticians as the *likelihood ratio* ($r$). In the case of a positive result, this ratio is:

$$r(R)=\frac{1-\beta}{\alpha} \quad (1)$$

The numerator of this ratio is the power of the test $(1-\beta)$, which corresponds to the probability of obtaining a "true positive" result, *i.e.* of concluding that there is a difference (Event $R$) when the treatments do in fact differ (Event $D$). The denominator is the level of significance, ($\alpha$), which is the probability of a "false positive" result, *i.e.* of concluding that there is a difference (Event $R$) when the treatments do not in fact differ (Event $ND$).

For the case of a null result, the corresponding likelihood ratio is:

$$r(NR)=\frac{1-\beta}{\beta} \quad (2)$$

where the ratio is the probability of a "true null" result, divided by the probability of a "false null" result. Thus, in both cases, $r$ is the ratio of the probability of drawing a correct conclusion to the probability of drawing an incorrect one. A large value for both $r(R)$ and $r(NR)$ is desirable, and is ensured by choosing small values for both $\alpha$ and $\beta$. It should be emphasized, however, that within the classical interpretation of probability, one cannot relate these values for $r$ to the belief of the researchers in their own results. Instead, $r$ is an *a priori* quantity entirely determined by the choice of experimental design.

At the level of the clinical–scientific community, however, these same results obtained by an individual research team will be evaluated in a context which will be biased *against* the occurrence of Event $D$ (a true treatment difference). This bias should exist for the reasons discussed above: the principle of organized scepticism, and the trade-off between ethics and scientific rigor. We have proposed that the bias can be estimated using the Bayesian prior probability $p$, or prior odds, $p/q$. The degree to which this initial belief is changed after consideration of the conclusions from a particular newly-completed trial will depend on the weight of the evidence, together with the strength of the initial belief, and can be assessed quantitatively using Bayes' theorem. For the simple case of 2 possible outcomes, Bayes' theorem can be expressed as:

$$\text{Posterior odds}=r\ (\text{prior odds}) \quad (3)$$

where $r$ is the likelihood ratio, from Equation (1) or Equation (2). That is, if the trial yields a positive result, the posterior odds in favour of the positive result are given by:

$$\text{Posterior odds}_{P}=\left(\frac{1-\beta}{\alpha}\right)\left(\frac{p}{q}\right) \quad (4)$$

If a null conclusion is reached, the posterior odds in favour of a null result are:

$$\text{Posterior odds}_N = \left(\frac{1-\alpha}{\beta}\right)\left(\frac{q}{p}\right) \quad (5)$$

Thus the evidence, as expressed by the likelihood ratio, acts on the prior odds to change them by a multiplicative factor $r$ (see also Lee, 1971).

A more detailed discussion of Equation (3) is given in the Appendix. It should be noted that this equation describes an ideal situation, in which the change in opinion takes place in a rational manner, and no bias other than the one represented by the prior odds is present.

Typical values of $\alpha$ and $\beta$ are 0·05 and 0·2 (Gehan & Schneiderman, 1973) although the value of $\beta$ actually achieved is often not as small at this (Freiman *et al.*, 1978). As discussed above, a value of $p=0{\cdot}2$ may represent a minimum average estimate based on current experience. For these values of the parameters, we obtain:

$r\ (R)=16,\ r\ (NR)=4{\cdot}75,\ p/q=0{\cdot}25,\ q/p=4,$ and the posterior odds, given that a positive result has occurred, are 4:1. The corresponding posterior odds, given that a null result has occurred, are 19:1. Expressed in terms of probabilities rather than odds, a positive result would change the probability of a true treatment difference from 0·2 (prior) to 0·8 (posterior), while a null result would change the probability of no true treatment difference from 0·8 (prior) to 0·95 (posterior). If we define the impact of a result by the strength of the belief with which they are accepted, as measured by the magnitude of the posterior probability or the posterior odds (rather than by the change in probability caused by the result) then a null result has a greater impact in this case than a positive result. Although positive results do change disbelief (odds less than unity) into belief, the degree of acceptance is lower than that given to null results. This consequence holds also for other common designs and is insensitive to small variations in $\alpha$, $\beta$ and $p$, as illustrated by the curves shown in Fig. 1. These curves represent

FIGURE.—Dependence of the posterior probability of effectiveness or ineffectiveness of a new treatment, expressed as the posterior odds, as a function of the prior probability $p$. For further explanation, see text, and Equations (4), (5) and (6).

Examples

| $\alpha$ | $\beta$ | $r(R)$ | $r(NR)$ | $p_0$ |
|---|---|---|---|---|
| 0·01 | 0·05 | 95·00 | 19·80 | 0·31 |
| 0·05 | 0·10 | 18·00 | 9·50 | 0·42 |
| 0·05 | 0·20 | 16·00 | 4·75 | 0·35 |
| 0·10 | 0·50 | 5·00 | 1·80 | 0·38 |

posterior odds, rather than posterior probabilities, as a function of the prior probability of a true treatment difference. As mentioned above, both probabilities and odds are measures of belief; choice of the latter measure of impact enhances differences between large probabilities, but does not change the argument qualitatively.

The pair of curves representing the posterior odds, given that a positive or a null result was obtained, intersect at the point

$$p_0 = 1/(1 + \sqrt{r(R)/r(NR)}) \qquad (6)$$

This is also the point of intersection of the corresponding probability curves.

For all values of $p$ less than $p_0$, we find that positive results have less impact, as defined above, than null results. Values for $p_0$ for the curves in the figure are given in its legend, for values of $p$ of about 0·4 or less, null results will have greater impact than positive ones. As discussed earlier, a randomized trial is probably inappropriate if $p$ exceeds 0·2 on the basis of current experience. Thus, null results would usually be expected to have a greater impact; if a positive result is obtained, its relatively low credibility could tend to slow down its use in clinical practice. Indeed, more experimentation might be necessary to reach the point where the opinion of the clinical–scientific community would be reversed. But at this point, a randomized clinical trial may have ceased to be ethical. The absence of the confirmatory scientifically rigorous evidence such an additional trial might provide could result in a continuing delay of acceptance of a new therapy.

This reasoning leads to the prediction that positive results would be expected to have less impact on the clinical–scientific community than null results. It is, however, based on the assumption that the clinical–scientific community will make use of new results in a "rational" manner, according to Bayes' theorem. Whether this is the case is certainly open to question, and needs to be tested experimentally (Lee, 1971; Lyon & Slovic, 1975). In practice, it is very likely that positive results from a well organized clinical trial will have *more* impact on the clinical–scientific community than would be predicted from Bayes' theorem, primarily because of the strong pressure of the need to improve treatments. Such pressure can, for example, lead to the early publication of positive results, while null results may often not be published at all (Peto *et al.*, 1976). The neglect of prior probabilities that is fostered by this pressure can lead to a recurring situation of false hope followed by disillusionment; an initial enthusiasm for a positive result is replaced by disappointment when subsequent results are less impressive.

### THE NEED FOR NEW APPROACHES

This analysis reveals some intrinsic limitations of the clinical-trial mechanism, limitations which tend to frustrate its basic aims. The analysis hinges on 2 aspects. One is the ethical need to plan therapeutic trials with small prior probabilities of success: as a consequence, most results are null, in accord with expectation. The other is the scientific need to evaluate new results in a manner which takes into account prior probabilities. If prior probabilities are considered, according to Bayes' theorem, null results have a much greater impact than positive ones. If, on the other hand, the clinical–scientific community, under the pressure of the need for more effective treatments, disregards prior probabilities and the principle of organized scepticism, the consequence is a tendency to encourage false hopes.

Is it possible to improve the system without violating ethical constraints or discouraging innovation? The answer is clearly negative *as long as success is defined exclusively in terms of cure.* We propose that the key to a substantial improvement is to broaden the definition of success in a meaningful way. In order to do this, it is necessary to be aware of the different sets of values which are at work in the health-care system, and to include considerations of these values in the planning of clinical trials. A careful consideration of values should make it easier to reach some consensus on what should be considered "success", either by explicitly using a particular value system, or by reaching an "optimal compromise" between different value systems.

It is beyond the scope of this paper to discuss in detail the different value systems at work in health care, but our discussion would be incomplete without a tentative outline. We shall attempt to develop a preliminary approach to value analysis. Although this approach is not novel in other fields, where systems analysis has been applied extensively (World Health Organization, 1976; Laszlo, 1972; Churchman, 1968) its relevance to clinical trials has received little attention.

Efforts to improve health may be considered to involve at least 5 broad interacting dimensions:

(a) improvements in understanding pathological processes and the action of therapeutic agents ("explanatory" dimension);
(b) improvements in eliminating disease and extending life ("cure" dimension);
(c) improvements in reducing the disruptive and painful effects of diseases ("care" dimension);
(d) improvements in controlling diseases and promoting health at the population level ("prevention" dimension);
(e) improvements in socio-economic efficiency ("cost" dimension).

A value system could be regarded as a set of "relative weights" for these dimensions.

Success of an attempt to improve health should be defined in terms of each of these dimensions. We shall omit the "prevention" dimension from the following discussion because we are concerned with the typical oncology trial, which does not involve large populations. The remaining dimensions, however, are all directly involved in a clinical trial and its potential impact on each of them should be analysed when the trial is being designed.

The impact of a clinical trial on the *explanatory* dimension can only be measured in terms of variables which are appropriate to the particular biological model under investigation (*e.g.* measurements at the molecular, cellular, tissue or system level). Such measurements can often be performed on biopsy or blood samples with little or no additional risk for the patients, and can provide important information about the natural history of the disease, or the effects of treatment. The successful acquisition of such information need not be dependent on improvements in other dimensions. A trial designed to shed further light on a biological problem was termed "explanatory" by Schwartz & Lellouch (1967). Their distinction between "explanatory" and "pragmatic" trials is useful in that it serves to emphasise the importance of making the appropriate distinctions at the planning stage of a trial. An examination of the remaining dimensions will allow us to distinguish different "pragmatic" aims.

For appraising improvements affecting the *cure* dimension, the duration of survival or the time to recurrence of disease are appropriate outcome variables. Survival time has been used increasingly as the response criterion for therapeutic trials in oncology (Bardelli & Saracci, 1978) and not surprisingly the language and the methodology to describe and study survival curves are well developed. Thus, in a sense, the cure-orientated value system has been the predominant one in such trials. We suggest that trials based *entirely* on the cure dimension are extremely difficult to plan with a high prior probability of success. The emphasis on survival data should therefore be balanced by proper attention to other variables.

Appropriate variables for the *care* dimension are those that measure short-term efficacy of a treatment. Thus, an improvement in care could be defined in terms of reduced side effects, amelioration of symptoms, and restoration of physical or psycho-social function. Measures of "quality of life" have often been discussed, but are seldom used (Bardelli & Saracci, 1978). The development of instruments such as those used to obtain health-status indices (Sackett & Torrance, 1978; Culyer, 1978; Kaplan *et al.*, 1976) may provide a useful approach to the assessment of such short-term variables.

Finally, variables of interest for the "*cost*" dimension would involve various types of cost (direct and indirect costs to the patient, to the health agencies, to society, *etc.*). By combining effectiveness and cost measures, comparisons of alternative approaches to health delivery could be performed. The techniques of cost-effectiveness analysis are still under development (Weinstein & Stason, 1977; Pliskin & Taylor, 1977) and, of course, are subject to the combined uncertainties of those involved in the assessment both of effectiveness and of cost.

A value analysis of the type outlined could help in the planning of clinical trials. While success along the cure dimension may remain unlikely, appropriate planning could select one or more of the other dimensions (depending on the value systems involved) about which an important question could be asked. It is difficult to imagine a trial which would have low prior probability of success along *all* the dimensions outlined above; a basic aim of the value analysis approach outlined above is to design clinical trials so that null results along all the dimensions selected would be rare.

### *An example: adult acute myeloid leukaemia (AML)*

A single example will be considered briefly in order to illustrate some of the points outlined in the previous section. The basis for the example is a trial on the chemotherapy of AML in adults (Medical Research Council, 1979). The clinical expertise involved in this trial was outstanding, as was the care taken in defining and following the protocol, and the thoroughness of the statistical analysis. The trial is chosen as representative of the clinical-trials mechanism, not in order to subject it to criticism but to use it to illustrate how a value analysis at the planning level might be helpful in obtaining additional information from such trials.

The purpose of this particular trial was to compare 2 regimens of multiple-drug chemotherapy, to see whether the extra toxicity expected from the more intensive regimen (TRAP) would be compensated for by higher remission rates and better survival. The overall results showed no significant difference between the 2 regimens in remission rate or duration of survival, although improvement in survival associated with more intensive chemotherapy was substantial for patients who had favourable prognostic features at presentation. From the viewpoint of value analysis, several points of interest about this trial become apparent:

(a) *Explanatory dimension.*—The rationale behind the use of more intensive therapy was that chemotherapy induces remission by killing leukaemic cells, and more intensive therapy should leave fewer surviving cells. A direct test of this rationale would require studies at the cellular level, designed to assess the effects of chemotherapeutic agents on the relevant cell populations. A provocative alternative view is that chemotherapeutic agents act at least in part by stimulating the differentiation of cells belonging to the leukaemic cell population (Sachs, 1978). Measurements at the cellular level designed to assess the explanatory dimension were not included in the MRC trial. However, cell-culture methodology for assessing sensitivity to chemotherapeutic agents is becoming available for leukaemic cells with proliferative potential (Buick *et al.*, 1979; Dicke *et al.*, 1976) and for clonogenic cells from solid tumours (Salmon *et al.*, 1978). A value system which gave major weight to the explanatory dimension would lead to the incorporation of studies at the cellular level into the design of a trial.

(b) *Cure dimension.*—This dimension received major emphasis in the design of the MRC trial, since the aim of the trial was to test whether or not more intensive therapy would improve remission frequency, duration of remission, or survival. The prior probability of a positive result is difficult to assess in retrospect, but was probably not unusually low; indeed, more intensive remission-induction therapy had already been reported to give high remission rates (see, *e.g.*, Gale & Cline, 1977). However, a null result was obtained in the MRC trial, in that the overall results showed no significant difference between the 2 protocols in remission rate or in duration of survival, although patients randomized to the more intensive regimen fared slightly better ($P = 0{\cdot}06$). However, retrospective stratification of the groups into categories of patients differing in important prognostic factors (such as age) indicated a superiority of the more intensive regimen for patients with favourable prognostic features. It is noteworthy that the results for *neither* of the regimens used in the MRC trial were as good as in the original reports (MRC, 1979). If Bayes' theorem were used to take these new results into account, they would be expected to have considerable impact, and might arouse scepticism about the probability of success of more intensive remission-induction therapy (see also Curtis *et al.*, 1979). However, it seems more likely that the null result to the question actually asked, on a prospective basis, in the trial ("Does more intensive therapy give better overall results?") will receive less weight in the clinical-scientific community than the question asked on a retrospective basis ("Do different categories of patients respond differently to more intensive therapy?").

(c) *Care and cost dimensions.*—The care and cost dimensions were not explicitly taken into account in the design of the trial, except to add specialized care for patients receiving more aggressive therapy. Yet in the report these dimensions are mentioned because they are important for a proper interpretation of the results. To quote the authors: "for the poor risk groups it seems likely that improvement in supportive care during remission induction therapy would reduce the risk of early death . . . but while supportive care is inadequate, more intensive therapy offers no advantage and its use is difficult to justify because of the extra toxicity, extra cost and the high incidence of side effects."

The above remarks would have a much greater practical weight if they could be supported by appropriate measurements of "toxicity", "side effects" and "cost". Of course, these measurements would need to be considered at the design stage; the relevant measurement techniques are still under development, as mentioned previously.

We conclude from this example of value analysis that consideration of the different dimensions involved in health care is feasible in the context of realistic clinical trials. It does not seem unreasonable to suggest that greater attention be paid to these dimensions in the design of future trials. Even though major attention will undoubtedly continue to be paid to the cure dimension, the incorporation into trials of measurements designed to answer explicit questions about other dimensions should greatly enhance the value or impact of such trials, even if the answers to the question based on the cure dimension turn out to be null.

This work was supported by the National Cancer Institute of Canada, the Ontario Cancer Treatment and Research Foundation, and the Ontario Ministry of Health. The authors are grateful to their colleagues at the Ontario Cancer Institute for many helpful comments.

### APPENDIX

In a comparison of treatments in a clinical trial, 4 events are of interest; they are represented by the symbols $R$, $NR$, $D$ and $ND$:

| | *Yes* | *No* |
|---|---|---|
| A difference is reported | $R$ | $NR$ |
| A true difference exists | $D$ | $ND$ |

*Before* the clinical trial, the prior probability (as a measure of belief) that $D$ is the case is:

$$P(D) = p \qquad \text{(A1)}$$

Also:

$$P(ND) = 1 - p = q \qquad \text{(A2)}$$

The design of the trial involves 4 probabilities:

$$P(R/D) = 1-\beta \quad \text{(A3)}$$

$$P(NR/D) = \beta \quad \text{(A4)}$$

$$P(R/ND) = \alpha \quad \text{(A5)}$$

$$P(NR/ND) = 1-\alpha \quad \text{(A6)}$$

For instance, $P(R/D)$, the probability of $R$ given $D$, is the probability of correctly concluding that a difference exists. It is given by an underlying probability model and can be identified with $1-\beta$, as in equation A3, where $\beta$ is the probability of missing a true difference (type II error).

*After* the experiment, either event $R$ or event $NR$ occurs. If $R$ is the case, then the posterior probability (again a measure of belief) is, by the definition of conditional probability

$$P(D/R) = \frac{P(RD)}{P(R)} = \frac{P(RD)}{P(D)} \cdot \frac{P(D)}{P(R)}$$

$$= P(R/D) \cdot \frac{P(D)}{P(R)} \quad \text{(A7)}$$

On the other hand,

$$P(R) = P(R/D) \cdot P(D) + P(R/ND) \cdot P(ND)$$
$$= p \cdot P(R/D) + q \cdot P(R/ND) \quad \text{(A8)}$$

Substitution in (A7) yields:

$$P(D/R) = \frac{p \cdot P(R/D)}{p \cdot P(R/D) + q \cdot P(R/ND)} \quad \text{(A9)}$$

If the $NR$ is the case, an analogous argument leads to:

$$P(ND/NR) = \frac{q \cdot P(NR/ND)}{q \cdot P(NR/ND) + p \cdot P(NR/D)} \quad \text{(A10)}$$

Equations A9 and A10 are the usual formulation of Bayes' theorem, applied to the 4 events of interest in a clinical trial. $P(D/R)$ and $P(ND/NR)$ are the posterior probabilities that the true situation coincides with what is reported for the 2 possible events $R$ and $NR$, *i.e.*, the posterior probabilities that a true positive or a true null result are obtained.

Also:

$$P(ND/R) = 1 - P(D/R) \quad \text{(A11)}$$

$$P(D/NR) = 1 - P(ND/NR) \quad \text{(A12)}$$

From (A9), (A10), (A11) and (A12):

$$\frac{P(D/R)}{P(ND/R)} = \frac{p \cdot P(R/D)}{q \cdot P(R/ND)} \quad \text{(A13)}$$

$$\frac{P(ND/NR)}{P(D/NR)} = \frac{q \cdot P(NR/ND)}{p \cdot P(NR/D)} \quad \text{(A14)}$$

Define:

$$\text{Posterior odds}_{\text{P}} = P(D/R)/P(ND/R) \quad \text{(A15)}$$

$$\text{Posterior odds}_{\text{N}} = P(ND/NR)/P(D/NR) \quad \text{(A16)}$$

$$\text{Prior odds}_{\text{P}} = p/q \quad \text{(A17)}$$

$$\text{Prior odds}_{\text{N}} = q/p \quad \text{(A18)}$$

$$r(R) = P(R/D)/P(R/ND) \quad \text{(A19)}$$

$$= (1-\beta)/\alpha, \text{ from (A3) and (A5)}$$

$$r(NR) = P(NR/ND)/P(NR/D) \quad \text{(A20)}$$

$$= (1-\alpha)/\beta, \text{ from (A4) and (A6)}$$

Then, from (A13) and A14):

$$\text{Posterior odds}_{\text{P}} = r(R) \cdot \text{Prior odds}_{\text{P}} \quad \text{(A21)}$$

$$\text{Posterior odds}_{\text{N}} = r(NR) \cdot \text{Prior odds}_{\text{N}} \quad \text{(A22)}$$

Or, in condensed form:

$$\text{Posterior odds} = r \cdot \text{Prior odds} \quad \text{(A23)}$$

### REFERENCES

BAILEY, K. D. (1978) *Methods of Social Reaserch.* New York: Free Press. p. 355.

BARDELLI, D. & SARACCI, R. (1978) Measuring the quality of life in cancer clinical trials: A sample survey of published trials. In *Methods and Impact of Controlled Therapeutic Trials in Cancer, Part I.* Ed. Flamant. Geneva: International Union Against Cancer. p. 75.

BUICK, R. N., MESSNER, H. A., TILL, J. E. & MCCULLOCH, E. A. (1979) Cytotoxicity of adriamycin and daunorubicin for normal and leukemic progenitor cells of man. *J. Natl Cancer Inst.*, **62,** 75.

BYAR, D. P., SIMON, R. M., FRIEDEWALD, W. T. & 5 others (1976) Randomized clinical trials: Perspectives on some recent ideas. *N. Engl. J. Med.*, **295,** 74.

CHALMERS, T. C., BLOCK, J. B. & LEE, S. (1972) Controlled studies in clinical cancer research. *N. Engl. J. Med.*, **287,** 75.

CHURCHMAN, C. W. (1968) *The Systems Approach.* New York: Delacourt Press. p. 179.

CULYER, A. J. (1978) *Measuring Health: Lessons for Ontario.* Toronto: University of Toronto Press. p. 58.

CURTIS, J. E., TILL, J. E., MESSNER, H. A., SOUSAN, P. & MCCULLOCH, E. A. (1980) Comparison of outcomes and prognostic factors for two groups of patients with acute myeloblastic leukemia. *Leukemia Res.* (in press).

D'ANGIO, G. J., CLATWORTHY, H. W., EVANS, A. E., NEWTON, W. A., JR & TEFFT, M. (1978) Is the risk of morbidity and rare mortality worth the cure? *Cancer*, **41**, 377.

DICKE, K. A., SPITZER, G. & AHEARN, M. J. (1976) Colony formation *in vitro* by leukaemic cells in acute myelogenous leukaemia with phytohaemagglutinin as stimulating factor. *Nature*, **259**, 129.

FEINSTEIN, A. R. (1977) *Clinical Biostatistics*. St Louis: C. V. Mosby. p. 320.

FHANER, S. (1977) Subjective probability and everyday life. *Scand. J. Psychol.*, **18**, 81.

FLAMANT, R. (1972) *Controlled Therapeutic Trials in Cancer*. Geneva: International Union Against Cancer. p. 7.

FREIMAN, J. A., CHALMERS, T. C., SMITH, H., JR & KEUBLER, R. R. (1978) The importance of beta, the type II error and sample size in the design and interpretation of the randomized control trial: Survey of 71 "negative" trials. *N. Engl. J. Med.*, **299**, 690.

GALE, R. P. & CLINE, M. J. (1977) High remission induction rate in acute myeloid leukaemia. *Lancet*, i, 497.

GEHAN, E. A. & SCHNEIDERMAN, M. A. (1973) Experimental design of clinical trials. In *Cancer Medicine*. Eds Holland & Frei. Philadelphia: Lea & Febiger. p. 499.

GILBERT, J. P., MCPEEK, P. & MOSTELLER, F. (1977) Statistics and ethics in surgery and anaesthesia. *Science*, **198**, 684.

KAPLAN, R. M., BUSH, J. W. & BERRY, C. C. (1976) Health status: Types of validity and the index of well-being. *Health Serv. Res.*, **11**, 478.

KEMPTHORNE, O' (1977) Why randomize? *J. Stat. Plan. Infer.*, **1**, 1.

LASZLO, E. (1972) *The Relevance of General Systems Theory*. New York: Braziller.

LEE, W. (1971) *Decision Theory and Human Behavior*. New York: Wiley. p. 250.

LYON, D. & SLOVIC, P. (1975) Dominance of individuality information and neglect of base rates in probability estimation. *Oregon Res. Inst. Bull.*, **15**, 18.

MARANELL, G. M. (1974) *Scaling: A Sourcebook for Behavioral Scientists*. Chicago: Aldine. p. 1.

MEDICAL RESEARCH COUNCIL (1979) Chemotherapy of acute myeloid leukemia in adults. *Br. J. Cancer*, **39**, 69.

MERTON, R. K. (1973) The normative structure of science. In *The Sociology of Science*. Chicago: University Press. p. 267.

MIKÉ, V. & GOOD, R. A. (1977) Medical research: Statistics and ethics. *Science*, **198**, 677.

PETO, R., PIKE, M. C., ARMITAGE, P. & 7 others (1976) Design and analysis of randomized clinical trials requiring prolonged observation of each patient. I. Introduction and design. *Br. J. Cancer*, **34**, 585.

PETO, R., PIKE, M. C., ARMITAGE, P. & 7 others (1977) Design and analysis of randomized clinical trials requiring prolonged observation of each patient. II. Analysis and examples. *Br. J. Cancer*, **35**, 1.

PLISKIN, N. & TAYLOR, A. K. (1977) General principles: Cost-benefit and decision analysis. In *Costs, Risks and Benefits of Surgery*. Eds Bunker, Barnes & Mosteller. New York: Oxford University Press. p. 5.

SACHS, L. (1978) Control of normal cell differentiation and the phenotypic reversion of malignancy in myeloid leukaemia. *Nature*, **274**, 535.

SACKETT, D. L. & TORRANCE, G. W. (1978) The utility of different health states as perceived by the general public. *J. Chron. Dis.*, **1**, 697.

SALMON, S. E., HAMBURGER, A. W., SOEHNLEN, B., DURIE, B. G. M., ALBERTS, D. S. & MOON, T. E. (1978) Quantitation of differential sensitivity of human-tumor stem cells to anti cancer drugs. *N. Engl. J. Med.*, **298**, 1321.

SCHWARTZ, D. & LELLOUCH, J. (1967) Explanatory and pragmatic attitudes in therapeutic trials. *J. Chron. Dis.*, **20**, 637.

TVERSKY, A. & KAHNEMAN, D. (1974) Judgment under uncertainty: Heuristics and biases. *Science*, **185**, 1124.

WEINSTEIN, M. C. & STASON, W. B. (1977) Foundations of cost-effectiveness analysis for health and medical practices. *N. Engl. J. Med.*, **296**, 716.

WORLD HEALTH ORGANIZATION (1976) *Application of Systems Analysis to Health Management*. Technical Report Series **596**. Geneva: World Health Organization. p. 6.